# Dasatinib-induced nephrotic syndrome: a case of phenoconversion?

**DOI:** 10.3325/cmj.2019.60.250

**Published:** 2019-06

**Authors:** Inga Mandac Rogulj, Vid Matišić, Borna Arsov, Luka Boban, Alen Juginović, Vilim Molnar, Dragan Primorac

**Affiliations:** 1 Department of Hematology, Clinical Hospital Merkur, Zagreb, Croatia; 2 University of Zagreb School of Medicine, Zagreb, Croatia; 3 School of Medicine, University of Split, Split, Croatia; 4 St. Catherine Specialty Hospital, Zabok/Zagreb, Croatia; 5 Eberly College of Science, The Pennsylvania State University, University Park, PA, USA; 6 School of Medicine, Josip Juraj Strossmayer University of Osijek, Osijek, Croatia; 7 Faculty of Dental Medicine and Health Osijek, Josip Juraj Strossmayer University of Osijek, Osijek, Croatia; 8 Faculty of Medicine, University of Rijeka, Rijeka, Croatia; 9 Henry C. Lee College of Criminal Justice and Forensic Sciences, University of New Haven, West Haven, CT, USA; 10 Children’s Hospital Srebrnjak, Zagreb, Croatia

## Abstract

We present the case of a 33-year-old chronic myeloid leukemia (CML) female patient, in whom the occurrence of nephrotic syndrome, during the treatment with tyrosine kinase activity inhibitors (TKIs), was potentially influenced by transient phenoconversion. Seven years after the CML diagnosis in 2004 and complete response, the patient experienced pain in the mandible and extremities. After this, imatinib was replaced by nilotinib, but generalized maculopapular rash was presented and successfully treated with antihistamines. The therapy was then discontinued due to planned pregnancy, and the patient experienced a relapse of CML with BCR-ABL/ABL1 transcripts of 18.9%. Dasatinib was introduced, and CML was in remission. Two years later, urine protein levels (6.19 g/L) and erythrocyte sedimentation rate were elevated (ESR = 90 mm/3.6 ks). The patient was diagnosed with nephrotic syndrome. With dasatinib dose reduction, urine protein level returned to the reference range. In order to determine the best genotype-guided therapy, the patient underwent pharmacogenomic testing, showing a homozygous CYP3A4 genotype *1/*1, associated with extensive metabolizer (EM) enzyme phenotype, typical for normal rates of drug metabolism for TKIs. However, this was inconsistent with nephrotic syndrome occurrence. A possible explanation would be CYP3A4 EM genotype coding a poor metabolizer enzyme phenotype, leading to the drug accumulation in the patient’s blood. This transient phenoconversion can be explained by inflammation with elevated ESR during nephrotic syndrome. This case shows that a broader approach that recognizes genetic, clinical, and epigenomic factors is required for a quality decision on the personalized therapy regimen.

We present the case of a 33-year-old chronic myeloid leukemia (CML) female patient, in whom nephrotic syndrome, during the treatment with tyrosine kinase activity inhibitors (TKIs), was potentially influenced by transient phenoconversion. Given the variable response to TKIs in patients with CML and constant dilemmas related to adverse drug reactions (ADRs) to TKIs, this case highlights a huge potential of pharmacogenomics in making optimal therapeutic decisions, even in the case of poor drug tolerability that otherwise may prompt physicians to stop the therapeutic regimen and jeopardize the treatment outcome. In addition, in this case report, we discuss the adverse impact of phenoconversion on the success of genotype-based prescribing decisions.

## Case presentation

The patient was diagnosed with CML in June 2004, after which she immediately started TKI therapy and had complete hematological, cytogenetic, and deep molecular response to therapy, but at a cost of persistent adverse drug reactions.

Seven years after the CML diagnosis and complete response, she experienced pain in the mandible and extremities. Imatinib was replaced by nilotinib, but generalized maculopapular rash was presented and successfully treated with antihistamines. The therapy was then discontinued due to planned pregnancy, and the patient experienced a relapse of CML with BCR-ABL/ABL1 transcripts of 18.9%. Dasatinib was introduced, and CML was in remission. Two years later, urine protein levels (6.19 g/L) and erythrocyte sedimentation rate were elevated (ESR = 90 mm/3.6 ks). The patient was diagnosed with nephrotic syndrome, and clinical investigation was conducted in order to determine its etiology. At that point, the patient did not have any signs of infection and did not take any nonsteroidal anti-inflammatory drugs or any other concomitant medication. She was examined by a nephrologist, who did not find indication for renal biopsy because drug-induced proteinuria was presumed. With dasatinib dose reduction, urine protein levels were returned to the reference range. In order to determine the best genotype-guided therapy, the patient underwent pharmacogenomic testing. The analysis showed that she had a homozygous CYP3A4 genotype *1/*1, associated with extensive metabolizer (EM) enzyme phenotype, typical for normal rates of drug metabolism for TKIs. However, this was inconsistent with nephrotic syndrome occurrence.

A possible explanation would be that CYP3A4 EM genotype coded a poor metabolizer (PM) enzyme phenotype, which led to the drug accumulation in the patient’s blood. This transient phenoconversion can be explained by inflammation with elevated ESR during nephrotic syndrome. The complete list of TKIs in the patient’s therapy, as well as all the adverse events associated with their usage, is shown in Table 1.

**Table 1:** The list of kinase activity inhibitors (TKIs) in the patient’s therapy and all the adverse events associated with their usage*

Therapy	Important date	Intervention	Adverse effects	Hematologic and urine parameters	Additional remarks
**Glivec (imatinib)**	November 2004	400 mg therapy introduction		Diagnosis of CML	
	May 2006			complete hematologic, cytogenetic and molecular response	
	June 2011		intensifying pain in the extremities and the mandible		NSAIDs did not reduce pain
	July 2011	therapy cessation			pain withdrew 2 weeks after discontinuation of the drug
**Tasigna (nilotinib)**	January 2012	800 mg therapy introduction	generalized maculopapular rash after a few days; successfully treated by antihistamines	complete hematologic, cytogenetic and molecular response	
	February 2012	therapy cessation			
	February 2012	400 mg therapy introduction		complete hematologic, cytogenetic and molecular response	dose reduction was done by the patient herself
	March 2012		generalized maculopapular rash successfully treated by antihistamines		
	October 2012	therapy cessation	generalized maculopapular rash successfully treated by antihistamines		
	October 2012	200 mg therapy introduction	generalized maculopapular rash treated by antihistamines	complete hematologic, cytogenetic and molecular response	
	May 2014	therapy cessation			the therapy was discontinued due to pregnancy planning
	November 2014	800 mg therapy introduction	generalized maculopapular rash and swelling of thighs unsuccessfully treated by antihistamines and corticosteroids	BCR-ABL/ABL1: 18.9% indicating a relapse of CML	
	December 2014	therapy cessation			the adverse events did not respond to the therapy, therefore it was decided to discontinue the therapy
**Sprycel (dasatinib)**	December 2014	100 mg therapy introduction		BCR-ABL/ABL1: 0.0058% indicating remission of CML	
	April 2016	therapy cessation	dry skin, periorbital and peripheral edema		the adverse events first happened over a year after therapy introduction
	April 2016	80 mg therapy introduction	dry skin, periorbital and peripheral edema, nausea and itching		therapy remained the same due to the patient’s ability to cope with adverse events
	November 2016				
	February 2017	therapy cessation	worsening of adverse effects, nephrotic-type proteinuria and elevated ESR	urine protein levels (UPL): 6.19 g/L† ESR: 90 mm/3.6 ks^‡^	
	February 2017	40 mg therapy introduction			improved general well-being of the patient
	March 2017			UPL: 1 g/L	
	July 2017			UPL: 1.12 g/L	
	October 2017	therapy cessation		ESR: 24 mm/3.6 ks Complete hematologic, cytogenetic and molecular remission	the therapy was discontinued due to pregnancy planning
**Bosulif (bosutinib)**	February 2018	500 mg therapy introduction	mandible pain and diarrhea following therapy introduction	increase in BCR-ABL/ABL1	the therapy was introduced due to CML remission
	May 2018	therapy cessation			
	May 2018	400 mg therapy introduction	mandible pain	8/2018 BCR-ABL/ABL1: MMR	the diarrhea had stopped after lowering the drug dose and remission was achieved
	August 2018	therapy cessation			
	August 2018	300 mg therapy introduction			confirmed major molecular response

*ESR – erythrocyte sedimentation rate; CML – chronic myeloid leukemia; NSAID – nonsteroidal anti-inflammatory drugs.

†reference range <0.2 g/L.

‡reference range = 4-24 mm/3.6 ks.

The patient was referred to the St. Catherine Hospital in September 2018 by her attending hematologist for pharmacogenomic testing, due to the broad spectrum of what were thought to be ADRs, and for determination of the best genotype-guided therapy. We tested the patient for polymorphisms in 25 known drug metabolizing enzymes and receptor coding genes (Table 2), as well as prothrombotic factors: factor II, V and methyltetrahydrofolate reductase (MTHFR), using RightMed^®^ panel (OneOme, Minneapolis, MN, USA). TaqMan real-time polymerase chain reaction (PCR) next-generation sequencing was performed to determine single nucleotide polymorphisms. The analysis showed that the patient had a homozygous CYP3A4 genotype *1/*1, associated with EM enzyme phenotype, typical for normal rates of drug metabolism. The patient and her attending hematologist were informed that the described ADRs were not a product of gene-drug interactions and advised that she should be guided as before, but with an emphasis on therapeutic drug monitoring for adverse events induced by either drug-drug interactions or by inflammation, to prevent future complications of TKI therapy. After the consultation with the patient’s hematologist, the patient continued bosutinib 200 mg per day with complete molecular and hematologic response without any debilitating adverse events.

**Table 2:** The list of genes responsible for the synthesis of enzymes involved in drug metabolism

Genes responsible for the synthesis of enzymes involved in the first phase of drug metabolism – cytochrome P450 enzymes	*CPY1A2*, *CYP2B6*, *CYP2C9*, *CYP2C19*, *CYP2C Cluster*, *CYP2D6*, *CYP3A4*, *CYP3A5*, *CYP4F2*
Genes responsible for the synthesis of enzymes involved in the second phase of drug metabolism	*TPMT*, *UGT1A1*
Genes responsible for the synthesis of other enzymes that are important for drug metabolism	*DPYD*, *VKORC1*, *NUDT15*
Genes responsible for the synthesis of drug transporters	*SLC6A4*, *SLCO1B1*
Genes responsible for the synthesis of drug receptors	*HTR2A*, *HTR2C*, *DRD2*, *OPRM1*, *GRIK4*, *COMT*
Genes responsible for the synthesis of other proteins important for drug function	*IFNL4*, *HLA-A*, *HLA-B*

## Discussion

The influence of the activity of CYP3A4 enzyme, the main enzyme metabolizing TKIs, on the pharmacokinetic profile of TKIs was previously reported (1). Therefore, we decided to test the patient’s genotype to determine her exact enzymatic activity. However, it is worth mentioning that recent studies presumed a link between ABCB1 expression and TKI metabolism *in vitro*, but the enzyme analysis was not included in the panel (2). The analysis showed that the patient had a homozygous CYP3A4 genotype *1/*1, associated with EM enzyme phenotype (3). The result was inconsistent with the observed adverse events, which could be explained by CYP3A4 genotype coding a PM enzyme phenotype, leading to the drug accumulation in the patient’s blood. Nephrotic syndrome did not appear immediately or soon after the drug administration, which would indicate a typical adverse drug reaction, but after a period of two years. To find an explanation for the case, we reviewed the literature on individualized pharmacotherapy led by genotypization.

Previous research on genotype-based individual pharmacotherapy demonstrated a series of mismatches between an enzyme’s genotype and predicted phenotype. This is most commonly attributed to enzyme inhibition caused by drug-drug interactions or a certain diet (4,5). A different approach to this problem would be to hypothesize that the elevation of proinflammatory cytokines (IL-1, IL-6, TNF-a), observed in conditions such as infections, diabetes, renal failure, or rheumatoid arthritis is capable of down-regulating the enzyme expression by mechanisms of intracellular signalization, as shown in *in vitro* and *in vivo* studies, alongside scarce clinical evidence (6,7). The described phenomenon is defined as phenoconversion, ie, conversion of genotypic EMs or intermediate metabolizers into phenotypic PMs of drugs, thereby modifying their clinical response to that of genotypic PMs (5).

Therefore, we hypothesize that the observed spectrum of adverse events, including nephrotic syndrome as the most prominent one, during dasatinib therapy in our patient was influenced by transient phenoconversion of CYP3A4 enzyme. One of the underlying mechanisms of this event is probably inflammation, as the adverse effect occurred in tandem with elevation of erythrocyte sedimentation rate 90 mm/3.6 ks, observed in the laboratory findings. The inflammation could have down-regulated cytochrome P450 enzymes, leading to dasatinib accumulation, which caused the adverse events.

Dasatinib has complex metabolism with five primary phase I metabolites, three of which are catalyzed by CYP3A4. The drug is a mechanism-based inactivator of CYP3A4 and it causes nicotinamide adenine dinucleotide phosphate (NADPH) and time-dependent CYP3A4 inactivation. This mechanism is unique to dasatinib compared to other TKIs. Previous results have implied that dasatinib is a mechanism-based inhibitor and has more serious drug-drug interactions than a typical competitive inhibitor (8). Dasatinib is unusually potent because the inactivation is cumulative and the enzymatic activity can only be restored after *de novo *protein synthesis. Li et al (8) have shown that hepatic accumulation of dasatinib or high intestinal and hepatic exposure during absorption may lead to higher than expected rates of CYP3A4 inactivation.

Moreover, it is important to underline that dasatinib could injure podocyte and endothelial cells through the inhibition of vascular endothelial growth factor, which may cause proteinuria and renal thrombotic microangiopathy (9). A considerable number of patients developed proteinuria after having been treated with dasatinib (10-14). Alahmari et al (10) assessed the incidence rate of TKI-associated proteinuria according to the TKI subtype in 256 CML patients. Median follow-up duration was 31 months for 35 months for dasatinib (1-105 months). The incidence rate of proteinuria in dasatinib treated group was 29/1000 person-year, while it was 0 in imatinib, nilotinib, bosutinib, and ponatinib-treated groups after having excluded secondary causes of proteinuria. Not all patients underwent kidney biopsy (10). In our study, nephrotic syndrome abated when dasatinib dose was decreased to 40 mg per day. This strengthens our hypothesis that the drug had initially accumulated in the patient’s blood in doses higher than needed, and lower daily doses were sufficient to achieve and retain complete therapeutic response with fewer ADRs. This case gave us an insight into the complexity of the mechanics that determine the phenotype of drug metabolizing enzymes other than genotype.

The case stands to remind all those included in the pharmacogenomics-guided therapy process that genotype assessment of patient’s drug response is insufficient to provide complete and safe care alone. A broader approach that recognizes genetic, clinical, and epigenomic (environmental) factors is required to make a safe and evidence-based decision on the personalized therapy regimen, alongside further development of existing guidelines established by Clinical Pharmacogenetics Implementation Consortium (*https://cpicpgx.org/)* and any other to come. Further prospective clinical studies are required to expand our knowledge on the implication of drug metabolizing enzyme genotyping on therapeutic effectiveness and patient outcome.
